# Association between neutrophil-to-lymphocyte ratio and short-term all-cause mortality in patients with cerebrovascular disease admitted to the intensive care unit-a study based on the MIMIC-IV database

**DOI:** 10.3389/fmed.2024.1457364

**Published:** 2024-10-02

**Authors:** Lin Lin, Jingyue Yang, Wenning Fu, Xi Liu, Yumin Liu, Li Zou

**Affiliations:** ^1^Department of Neurology, Zhongnan Hospital of Wuhan University, Wuhan, Hubei, China; ^2^School of Nursing, Tongji Medical College, Huazhong University of Science and Technology, Wuhan, Hubei, China

**Keywords:** neutrophil-to-lymphocyte ratio, cerebrovascular disease, all-cause mortality, MIMIC-IV, inflammatory biomarker

## Abstract

**Background:**

Inflammation plays a crucial role in cerebrovascular disease (CVD) progression. Neutrophil-to-lymphocyte ratio (NLR) is an important inflammatory marker, though its diagnostic role in CVD is still under investigation. This study evaluates the relationship between NLR and short-term all-cause mortality in patients with CVD admitted to the intensive care unit (ICU).

**Methods:**

We conducted a retrospective study using data from the Medical Information Mart for Intensive Care (MIMIC-IV) (v2.2) database, including 4,327 adult ICU-admitted CVD patients. NLR values at admission were analyzed alongside various mortality variables. Multivariate Cox proportional hazards regression models and Kaplan–Meier (K-M) survival curves assessed the relationship between NLR and short-term all-cause mortality. Predictive power, sensitivity, specificity, and area under the curve (AUC) of NLR for short-term mortality were investigated using Receiver Operating Characteristic (ROC) analysis. Additionally, restricted cubic spline (RCS) curves and subgroup analyses were conducted.

**Results:**

Among the 4,327 patients, 3,600 survived (survival group) and 727 died (non-survival group) within 28 days of admission (mortality rate: 16.8%). A multivariate Cox regression analysis identified NLR as an independent predictor of 28-day all-cause mortality (hazard ratio: 1.013; 95% confidence interval: 1.0086–1.0188; *p* < 0.001). The predictive model, incorporating NLR, age, gender, BMI, Charlson comorbidity index (CCI), WBC counts, Platelet, INR, and CRP, achieved an AUC of 0.686 (95% confidence interval: 0.665–0.70). While platelet-to-lymphocyte ratio was also analyzed, its predictive efficiency was less pronounced compared to NLR. A best NLR threshold of 6.19 was determined, distinguishing survivors from non-survivors. Kaplan–Meier survival curves showed that patients with NLR ≥ 6.19 had significantly lower survival rates at 7-, 14-, 21-, and 28-days. Subgroup analyses indicated that NLR did not significantly interact with most subgroups.

**Conclusion:**

NLR may serve as an independent predictor for short-term all-cause mortality in ICU-admitted CVD patients, enhancing our understanding of the association between inflammatory biomarkers and CVD prognosis.

## 1 Introduction

Cerebrovascular diseases (CVD), which include ischemic stroke, hemorrhagic stroke, transient ischemic attack, aneurysm and cerebrovascular malformation, are among the most common and devastating conditions affecting the brain’s blood vessels (1). It is responsible for up to 12% of deaths worldwide, is a major cause of disability, and represents an area of real unmet clinical need, accounting for a significant proportion of the disease burden (2). In the intensive care unit (ICU), CVD patients often present with severe symptoms and complications, requiring immediate and intensive medical attention (3). The mortality rate for these patients remains high, with many succumbing to their condition or suffering long-term disabilities. Given the critical nature of their condition, it is essential to have reliable prognostic indicators to identify patients at high risk of adverse outcomes (4). These indicators can guide clinical decision-making, optimize treatment strategies, and ultimately improve patient outcomes in the ICU.

Inflammation plays a pivotal role in the pathogenesis of CVD, contributing to both the initiation and progression of these conditions. The intricate relationship between CVD and inflammation is increasingly recognized, with evidence suggesting that inflammatory processes may exacerbate the clinical manifestations of CVD, such as stroke and cognitive impairment (5). Furthermore, systemic inflammation has been linked to the development of CVD subtypes, highlighting the importance of considering inflammatory markers in the diagnosis and treatment of cerebrovascular disorders (6). The neutrophil-to-lymphocyte ratio (NLR) is a novel marker of inflammation that indicates the severity of an inflammatory response. An increase in neutrophils is associated with inflammation, while a decrease in lymphocytes is linked to the body’s stress. Research has shown that NLR is closely related to the prognosis of a range of diseases, including chronic obstructive pulmonary disease, ulcerative colitis, gastrointestinal stromal tumors, atherosclerosis, liver cancer, and pancreatic cancer, subtly affecting their onset, progression, and outcomes (7). Unlike complex scoring systems, NLR is quick, cost-effective, and easy to integrate into clinical practice.

While NLR has been studied as a predictor of outcomes in many cardio and cerebrovascular conditions, such as myocardial infarction, atherosclerotic stenosis, ischimic stroke (7–9). However, its link with short-term mortality in ICU patients with CVD is not well understood. Similarly, the platelet-to-lymphocyte ratio (PLR) is another inflammatory marker that has shown promise in various clinical settings. PLR, which combines platelet and lymphocyte counts, has been linked to outcomes in cardiovascular diseases and cancer, and may provide complementary insights when used alongside NLR (10).

In this study, using the comprehensive MIMIC-IV database (v2.2), we aim to investigate the association between NLR and short-term all-cause mortality in ICU patients with CVD. By comparing NLR with PLR, we seek to elucidate their relative prognostic value and potential clinical applications in this high-risk patient population.

## 2 Methods

### 2.1 Data source

The data for this study was obtained from the MIMIC-IV database (v2.2). It is a comprehensive and publicly accessible database containing deidentified health-related data from over 70,000 ICU admissions at the Beth Israel Deaconess Medical Center in Boston, Massachusetts, United States, between 2008 and 2019, by the Massachusetts Institute of Technology Computational Physiology Laboratory.^1^ To ensure patient privacy, all personal information was anonymized using random codes, eliminating the need for patient consent or ethical approval. This database is publicly accessible via the PhysioNet online platform.^2^ To access it, the vital author, Li Zou, completed the Collaborative Institutional Training Initiative (CITI) course and passed exams on “Conflict of Interest” and “Data or Sample Only Research” (ID: 13349610). Following this, our research team was granted authorization to use and extract data from the database.

### 2.2 Population selection and results

**Figure 1:**
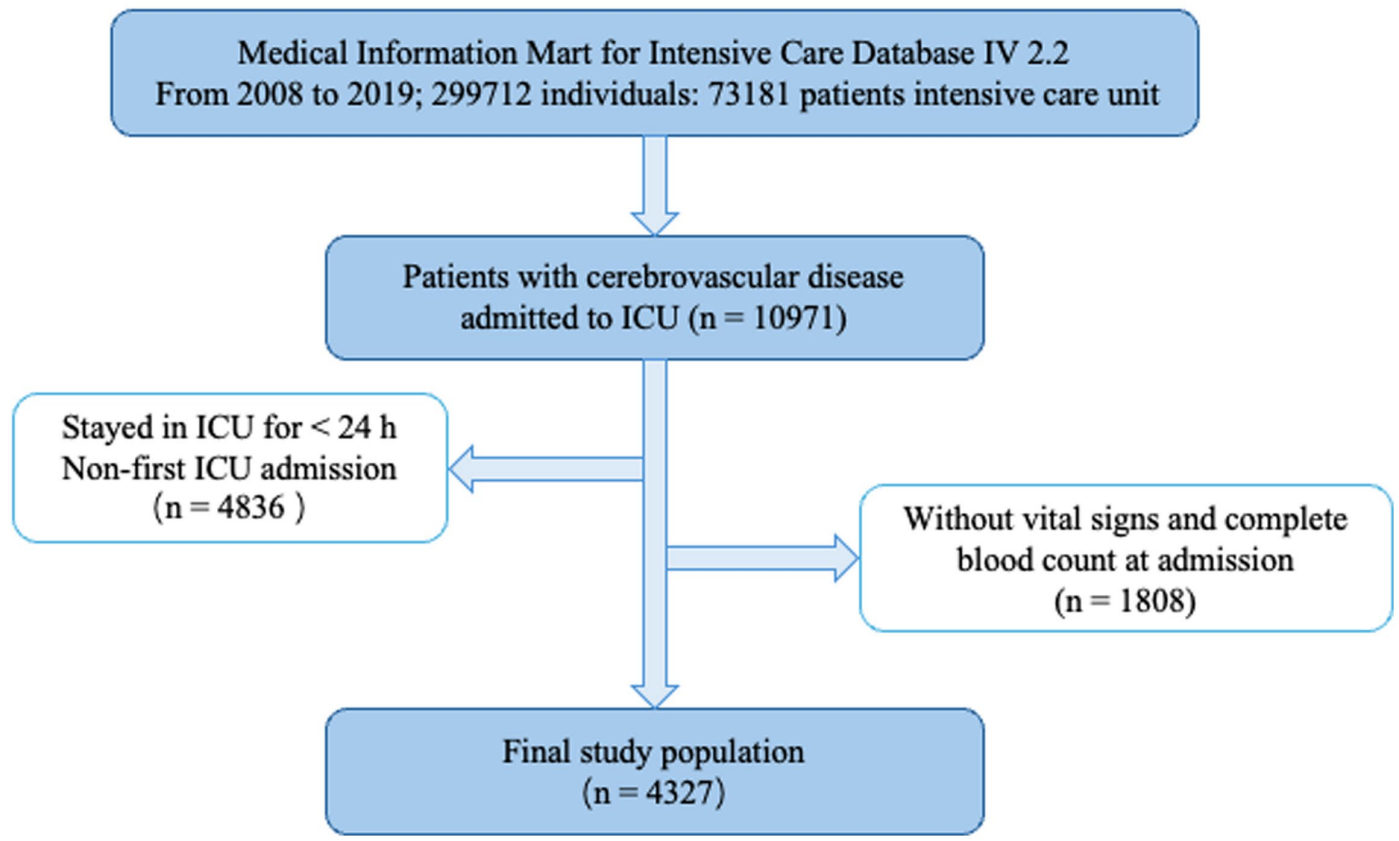
Flowchart for participants from the MIMC-IV (v2.2).

This study retrospectively included patients with cerebrovascular disease admitted to the ICU. The inclusion criteria were: (1) age above 18 years; (2) patients diagnosed with cerebrovascular disease according to ICD-9 and ICD-10 codes; and (3) patients who stayed in the ICU for at least 24 h. Patients with missing key data were excluded. For patients with multiple ICU admissions, only the first admission was considered. Finally, 4,327 patients were included in this study (Figure 1).

### 2.3 Data extraction

The primary exposure of interest was the NLR, a recognized marker of systemic inflammation. NLR was calculated as the ratio of the absolute neutrophil count to the absolute lymphocyte count, both obtained from the complete blood count at the first ICU admission. Additionally, we also investigated the PLR, another inflammatory marker that has shown prognostic value in various clinical settings. PLR was calculated as the ratio of the platelet count to the absolute lymphocyte count. Both NLR and PLR were included in our analysis to compare their prognostic performance.

The primary outcomes were in-hospital mortality and length of ICU stay. Mortality data, encompassing all-cause mortality within 7 days, 14 days, 21 days, 28 days, 90 days, and 1 year of admission, was obtained from hospital records. We considered various potential confounders, including demographic details, vital signs, comorbid conditions, laboratory parameters, and outcomes (Table 1). Data extraction was performed utilizing Postgres (v2.7.3) and pgAdmin4 (version 8.6) with structured query language.

**Table 1:** Covariates extracted in detail from the MIMIC-IV database.

Items	Composition
Demographic details	Age, gender, height, weight, body mass index
Vital signs	Systolic and diastolic blood pressure, heart rate, Glasgow coma scale
Comorbid conditions	Myocardial infarction, congestive heart failure, diabetes, chronic pulmonary disease, renal disease, rheumatic disease, peptic ulcer disease, liver disease (mild/severe), paraplegia, malignant cancer, metastatic solid tumor, Carlson comorbidity index (CCI)
Laboratory parameters	Red and white blood cell counts, lymphocytes, monocytes, neutrophils, platelet, blood urea nitrogen (BUN), Eosinophil counts, triglyceride (TC), D-dimer, fibrinogen, INR, PT, PTT, CRP
Clinical outcomes	ICU_Stays, Hospital_Stays, ICU mortality, In-hospital mortality, 7-day mortality, 14-day mortality, 21-day mortality, 28-day mortality, 90-day mortality, 1-year mortality

ICU, intensive care unit; BMI, body mass index; GCS, Glasgow Coma Scale; CCI, Charlson Comorbidity Index; WBC, white blood cell; RBC, red blood cell; BUN, blood urea nitrogen; TC, triglyceride; INR, international normalized ratio; T, prothrombin time; PTT, partial thromboplastin time; CRP, C-reactive protein.

### 2.4 Management of missing data and outliers

Variables with more than 15% missing values, such as blood urea nitrogen (BUN), basophils and eosinophil counts, triglyceride (TC); were omitted from the analysis to mitigate potential biases. For variables with less than 15% missing values, including lymphocyte, monocyte, neutrophil and platelet counts, multiple imputation prothrombin time (PT), international normalized ratio (INR), C-reactive protein (CRP), was performed to select the most appropriate dataset for imputing missing values. This approach ensured the integrity of the data and maintained the reliability of subsequent analyses.

### 2.5 Follow-up and endpoints

The follow-up duration for mortality outcomes was 1 year, with specific focus on short-term mortality at 7, 14, 21, 28, and 90 days. Follow-up data were obtained through hospital records, and the primary endpoint was all-cause mortality (more details). Survival time was calculated from the date of ICU admission to the date of death or the end of the follow-up period. The diagnostic criteria for study endpoints were based on standardized hospital protocols, ensuring consistency across cases.

### 2.6 Statistical analysis

Descriptive statistics were employed to summarize the baseline characteristics of the study cohort. Continuous variables were expressed as mean ± standard deviation (SD) or median (interquartile range [IQR]), while categorical variables were presented as frequencies and percentages. Continuous variables had been detected by normal distribution (Supplementary Table S1). Comparative analyses between groups utilized Student’s *t*-test or Mann–Whitney U test for continuous variables, and chi-squared test or Fisher’s exact test for categorical variables, as appropriate. NLR values were categorized into tertiles, with the highest tertile classified as high and the lower two tertiles as low. A univariate Cox regression analysis identified potential risk factors and a multivariate Cox regression analysis determined the independent risk factors for in-hospital mortality with *p*-values below 0.05. Potential confounders were identified based on prior literature and clinical relevance (10–12). These confounders included demographic factors, comorbidities, and critical illness scores. Confounders were adjusted for in the multivariate models to isolate the independent effect of NLR and PLR on outcomes. We performed collinearity checks by calculating the variance inflation factor (VIF) for each covariate, and the results indicated no significant multicollinearity (Supplementary Table S2). The prognostic performance of NLR for in-hospital mortality was evaluated using receiver operating characteristic (ROC) curves and calculating the area under the curve (AUC). The Youden index was utilized to determine the optimal cutoff value. Restricted cubic spline (RCS) analysis was employed to depict the non-linear relationship between NLR and short-term all-cause mortality in CVD subjects. Subgroup analyses were performed to examine the consistency of the findings across different patient populations. Subgroups were defined based on clinically significant variables. The rationale for choosing these subgroups was to explore whether the prognostic value of NLR and PLR varied across different patient characteristics. Survival distributions between subgroups were depicted using Kaplan–Meier survival curves, and differences were evaluated via log-rank tests. Statistical analyses were performed using R statistical software (version 4.3.2) and SPSS statistics software (version 29.0). A significance level of 0.05 was used for all hypothesis tests.

## 3 Results

### 3.1 Baseline demographic and clinical characteristics

Table 2 summarizes the baseline characteristics of the study population, which included 4,327 patients, of whom 2,294 (53%) were male, with a median age of 68 years (IQR: 58–78). The 28-day mortality rate was approximately 16.8%.

**Table 2:** Baseline characteristics between survivors within 28-day and non-survivors.

Variable	Total	Survivors	Non-Survivors	*p*
	(*n* = 4,327)	(*n* = 3,600)	(*n* = 727)	
Age, median (IQR)	68 (58, 78)	68 (57, 77)	75 (64, 83)	<0.001
Gender: male, *n* (%)	2,294 (53.02)	1919 (53.5)	375 (51.6)	0.415
Height, median (IQR), cm	168.0 (160.02, 176.53)	168.0 (160.02, 177.17)	168.2 (160.02, 175.26)	0.978
Weight, median (IQR), kg	78.5 (66.57, 92.7)	78.4 (66.51, 92.87)	79.4 (66.9, 92.30)	0.722
BMI, median (IQR)	27.53 (24.12, 31.67)	27.48 (23.95, 31.67)	27.71 (25.04, 31.56)	0.485
SBP, median (IQR), mmHg	124 (113, 136)	124 (114, 136)	125 (112, 137)	0.718
DBP, median (IQR), mmHg	64 (56, 73)	64 (56, 73)	63 (56, 71)	0.066
Heart Rate, median (IQR), bpm	80 (70, 90)	79 (70, 90)	80 (71, 91)	0.196
GCS, median (IQR)	15 (14, 15)	15 (14, 15)	15 (14, 15)	0.159
Myocardial infarct, *n* (%)	656 (15.16)	520 (12.02)	136 (3.14)	0.005
Congestive heart failure, *n* (%)	865 (19.99)	520 (12.02)	203 (4.69)	<0.001
Peripheral vascular disease, *n* (%)	706 (16.32)	662 (15.3)	96 (2.22)	0.013
Chronic pulmonary disease, *n* (%)	836 (19.32)	610 (14.1)	133 (3.07)	0.442
Rheumatic disease, *n* (%)	118 (2.73)	703 (16.25)	20 (0.46)	0.965
Peptic ulcer disease, *n* (%)	50 (1.16)	98 (2.26)	8 (0.18)	0.879
Mild liver disease, *n* (%)	224 (5.18)	42 (0.97)	67 (1.55)	<0.001
Severe liver disease, *n* (%)	69 (1.59)	157 (3.63)	29 (0.67)	<0.001
Diabetes, *n* (%)	1,229 (28.4)	40 (0.92)	197 (4.55)	0.417
Paraplegia, *n* (%)	792 (18.3)	1,032 (23.85)	196 (4.53)	<0.001
Renal disease, *n* (%)	701 (16.2)	596 (13.77)	142 (3.28)	0.008
Malignant cancer, *n* (%)	397 (9.17)	559 (12.92)	89 (2.06)	0.002
Metastatic solid tumor, *n* (%)	179 (4.14)	308 (7.12)	53 (1.22)	<0.001
Charlson comorbidity index, median (IQR)	5 (3, 7)	5 (3, 7)	6 (5, 8)	<0.001
RBC, median (IQR), 10^9/L	3.62 (3.12, 4.15)	3.63 (3.14, 4.16)	3.59 (3.04, 4.13)	0.052
WBC, median (IQR), 10^9/L	9 (6.8, 12.2)	8.8 (6.7, 11.7)	10.7 (7.7, 14.8)	<0.001
Lymphocytes, median (IQR), 10^9/L	1.41 (0.83, 2.23)	1.45 (0.86, 2.2575)	1.23 (0.73, 2)	<0.001
Monocytes, median (IQR), 10^9/L	0.51 (0.33, 0.76)	0.5 (0.33, 0.74)	0.57 (0.35, 0.85)	<0.001
Neutrophils, median (IQR), 10^9/L	6.53 (4.7, 9.2)	6.29 (4.5725, 8.74)	8.27 (5.69, 11.7)	<0.001
Platelet, median (IQR), 10^9/L	216 (165, 276)	216 (171, 281)	196 (134, 252)	<0.001
D-dimer, median (IQR), ug/mL	1,164 (536, 2,732)	1,163 (533, 2,732)	1,173 (554, 2,712)	0.592
Fibrinogen, median (IQR), mg/dL	293 (208, 430)	289 (207, 418)	322 (220, 463)	<0.001
INR, median (IQR)	1.1 (1.1, 1.3)	1.1 (1, 1.3)	1.2 (1.1, 1.4)	<0.001
PT, median (IQR)	12.6 (11.5, 14.2)	12.5 (11.4, 13.9)	13.1 (11.9, 15.2)	<0.001
PTT, median (IQR)	29.6 (26.5, 34.6)	29.6 (26.625, 34.3)	29.4 (26.1, 35.5)	0.301
CRP, median (IQR), mg/L	10.9 (2.8, 62)	11.1 (2.725, 60.7)	10.5 (2.9, 67)	0.976
PLR, median (IQR)	154.33 (91.7, 262.68)	154.08 (92.8, 260.94)	157.68 (81.97, 272.28)	0.37
NLR, median (IQR)	4.56 (2.55, 8.89)	4.26 (2.46, 8.17)	7.05 (3.19, 12.53)	<0.001

BMI, body mass index; SBP, systolic blood pressure; DBP, diastolic blood pressure; GCS, Glasgow Coma Scale; RBC, red blood cell; WBC, white blood cell; INR, international normalized ratio; PT, prothrombin time; PTT, partial thromboplastin time; CRP, C-reactive protein; PLR, platelet-to-lymphocyte ratio; NLR, neutrophil-to-lymphocyte ratio; IQR, interquartile range.

Non-survivors were significantly older (median age 75 vs. 68 years, *p* < 0.001). Non-survivors group also had a higher prevalence of myocardial infarction, congestive heart failure, peripheral vascular disease, Mild or severe liver disease, paraplegia, renal disease, malignant cancer, and metastatic solid tumors (all *p* < 0.05). The Charlson comorbidity index (CCI) was higher among non-survivors (median 5 vs. 6, *p* < 0.001).

Laboratory markers showed significant differences between the groups – non-survivors had higher white blood cell (WBC) counts, neutrophils, monocytes, INR, PT, fibrinogen, and NLR levels (all *p* < 0.001), as well as higher D-dimer (*p* = 0.592) and (*p* < 0.001) levels. They had lower lymphocytes, and platelets (all *p* < 0.001). Other covariates, including RBC and CRP, did not show significant differences between survivors and non-survivors groups (*p* > 0.05).

### 3.2 NLR is an independent risk factor for all-cause mortality within 28 days of hospital admission

In the univariate Cox regression analysis (Table 3), an unadjusted NLR showed a significant association with all-cause mortality within 28 days of admission [hazard ratio (HR), 1.015; 95% confidence interval (CI), 1.0100–1.0205; *p* < 0.001]. Our results indicate multiple factors were correlated with 28-day all-cause mortality, including age, congestive heart failure (HR, 1.378; 95% CI, 1.1718–1.6205; *p* < 0.001), mild liver disease (HR, 1.483; 95% CI, 1.1530–1.9074; *p* = 0.0021), severe liver disease (HR, 1.829; 95% CI, 1.2606–2.6537; *p* = 0.0015), paraplegia (HR, 1.5086; 95% CI, 1.2805–1.7772; *p* < 0.001), metastatic solid tumor (HR, 1.8385; 95% CI, 1.3899–2.4318; *p* < 0.001), CCI (HR, 1.1239; 95% CI, 1.0971–1.1513; *p* < 0.001), WBC counts (HR, 1.0114; 95% CI, 1.0079–1.0150; *p* < 0.001), neutrophil counts (HR, 1.0510; 95% CI, 1.0409–1.0611; *p* < 0.001), platelet counts (HR, 0.9967; 95% CI, 0.9960–0.9975; *p* < 0.001), and INR (HR, 1.1537; 95% CI, 1.0581–1.2578; *p* = 0.0012). Additionally, in these patients, the unadjusted platelet-to-lymphocyte ratio (PLR) also demonstrated significant correlation (HR, 0.9993; 95% CI, 0.9989–0.9996; *p* < 0.001).

**Table 3:** Univariate Cox regression models evaluating the association between NLR and 28-day all-cause mortality with CVD.

Variable	HR	95%CI	*p*
Age	1.0296	1.0239–1.0353	**<0.001**
Gender	0.8935	0.7725–1.0334	0.1293
Height	0.9997	0.9930–1.0065	0.9523
Weight	1.0001	0.9967–1.0036	0.9192
BMI	1.0013	0.9906–1.0122	0.8017
SBP	1.0014	0.9971–1.0056	0.5124
DBP	0.9953	0.9891–1.0016	0.1452
Heart Rate	1.0022	0.9978–1.0066	0.3245
GCS	0.9806	0.9532–1.0088	0.1772
Myocardial infarct	1.1976	0.9938–1.4431	0.0581
Congestive heart failure	1.3780	1.1718–1.6205	**<0.001**
Peripheral vascular disease	0.8932	0.7205–1.1073	0.3031
Chronic pulmonary disease	0.8790	0.7283–1.0608	0.1789
Rheumatic disease	1.1092	0.7111–1.7300	0.6476
Peptic ulcer disease	0.7494	0.3733–1.5045	0.4173
Mild liver disease	1.4830	1.1530–1.9074	**0.0021**
Severe liver disease	1.8290	1.2606–2.6537	**0.0015**
Diabetes	0.8552	0.7262–1.0072	0.0611
Paraplegia	1.5086	1.2805–1.7772	**<0.001**
Renal disease	1.1393	0.9484–1.3686	0.1633
Malignant cancer	1.2148	0.9731–1.5166	0.0855
Metastatic solid tumor	1.8385	1.3899–2.4318	**<0.001**
Charlson comorbidity index	1.1239	1.0971–1.1513	**<0.001**
RBC	1.1079	1.0018–1.2251	0.0459
WBC	1.0114	1.0079–1.0150	**<0.001**
Lymphocytes	1.0047	0.9963–1.0131	0.2689
Monocytes	1.1400	1.0346–1.2562	**0.0081**
Neutrophils	1.0510	1.0409–1.0611	**<0.001**
Platelet	0.9967	0.9960–0.9975	**<0.001**
D-dimer	1.0000	0.9999–1.0000	0.6562
Fibrinogen	1.0004	1.0000–1.0008	0.0145
INR	1.1537	1.0581–1.2578	**0.0012**
PT	1.0071	0.9992–1.0151	0.0760
PTT	0.9963	0.9921–1.0005	0.0863
CRP	0.9991	0.9980–1.0003	0.1535
PLR	0.9993	0.9989–0.9996	**<0.001**
NLR	1.0153	1.0100–1.0205	**<0.001**

BMI, body mass index; SBP, systolic blood pressure; DBP, diastolic blood pressure; GCS, Glasgow Coma Scale; RBC, red blood cell; WBC, white blood cell; INR, international normalized ratio; PT, prothrombin time; PTT, partial thromboplastin time; CRP, C-reactive protein; PLR, platelet to lymphocyte ratio; NLR, neutrophil to lymphocyte ratio.

**Figure 2:**
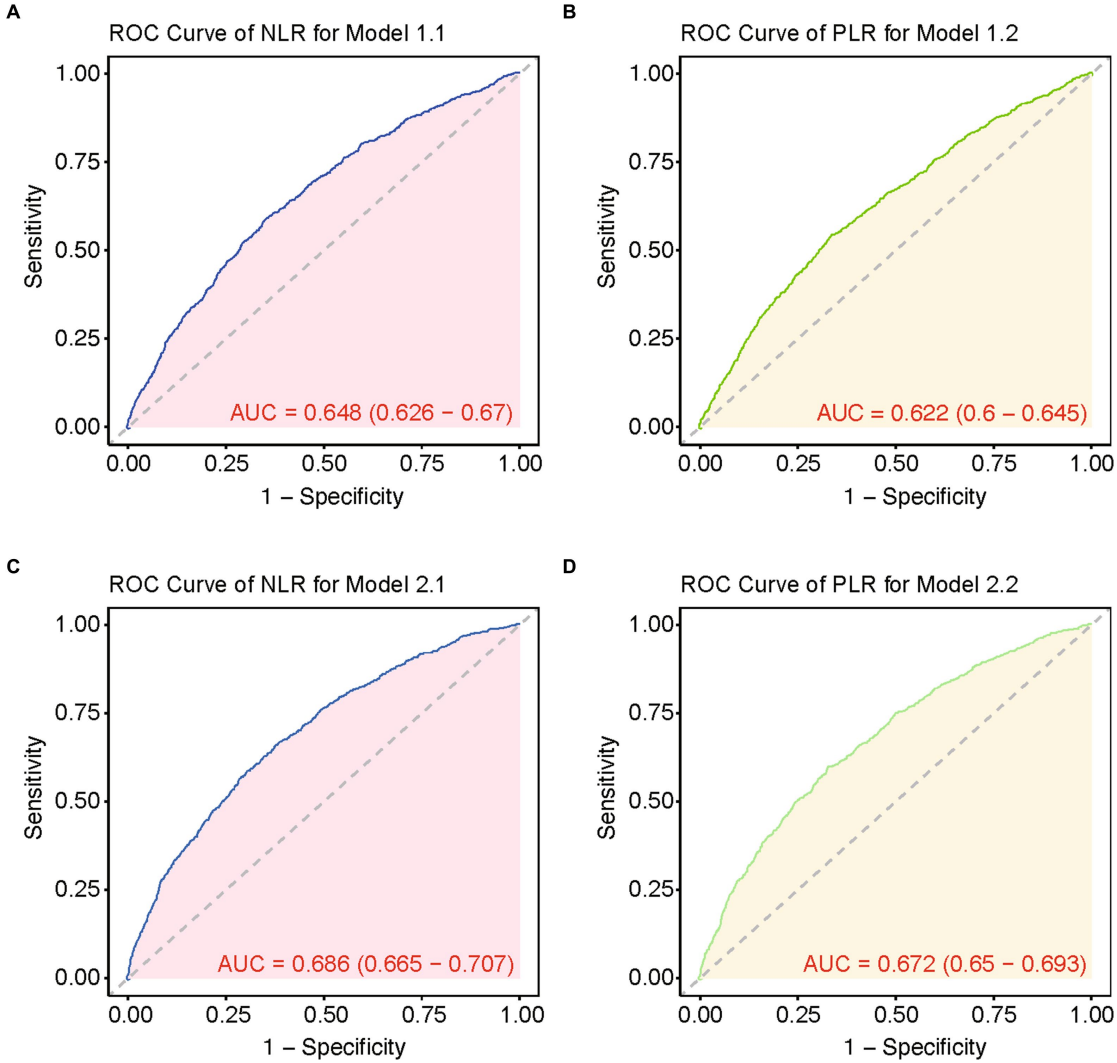
ROC curves of multiple models for predicting 28-day mortality. **(A)** ROC curve of NLR for model 1.1. **(B)** ROC curve of PLR for model 1.2. **(C)** ROC curve of NLR for model 2.1. **(D)** ROC curve of PLR for model 2.2.

Subsequently, we conducted the multivariate Cox regression analysis (Table 4). Our findings revealed that NLR remained significantly associated with higher in-hospital 28-day all-cause mortality, after adjusting for potential confounders such as age, gender, and body mass index (BMI) in Model 1.1 (HR, 1.016; 95% CI, 1.011–1.022; *p* < 0.001). Furthermore, in Model 2.1, which included additional adjustments for WBC counts, CCI, Platelet, INR and CRP, NLR continued to independently predict increased mortality risk (HR, 1.013; 95% CI, 1.0086–1.0188; *p* < 0.001). While PLR showed statistical significance in Model 1.2 (*p* = 0.001) like NLR, it did not retain statistical significance in Model 2.2 after adjusting for additional variables including WBC, CCI, platelet counts, INR, and CRP (*p* = 0.678). The overall performance of these models is illustrated in Figure 2. It displays that the ROC curves for predicting all-cause mortality within 28 days after admission of CVD patients showed that Model 1.1 achieved an area under the curve (AUC) of 0.648 (95% CI, 0.626–0.67), with a sensitivity of 60.37% and specificity of 64.50%; Model 2.1 had an AUC of 0.686 (95% CI, 0.665–0.707), sensitivity of 65.61%, and specificity of 62.08%. Comparatively, Model 1.1 exhibited an AUC of 0.622 (95% CI, 0.6–0.645), sensitivity of 54.07%, and specificity of 66.13%; Model 2.2 showed an AUC of 0.672 (95% CI, 0.65–0.693), sensitivity of 61.51%, and specificity of 67.2%.

**Table 4:** Multivariable Cox regression models evaluating the association between NLR/PLR and 28-day all-cause mortality with CVD.

Variable	HR	95%CI		*p*
*Model 1.1*				
NLR	1.016	1.011	1.022	**<0.001**
Age	1.029	1.024	1.036	**<0.001**
Gender	0.975	0.842	1.129	0.738
BMI	0.999	0.988	1.010	0.946
*Model 1.2*				
PLR	0.999	0.9991	0.9998	0.001
Age	1.029	1.0233	1.0348	**<0.001**
Gender	0.959	0.8288	1.1107	0.579
BMI	1.000	0.9897	1.0115	0.92
*Model 2.1*				
NLR	1.013	1.0086	1.0188	**<0.001**
Age	1.022	1.0162	1.0295	**<0.001**
Gender	0.894	0.7704	1.0375	0.140
BMI	0.998	0.9877	1.0097	0.807
WBC	1.008	1.0049	1.0119	**<0.001**
Charlson comorbidity index	1.048	1.0171	1.0818	0.002
Platelet	0.997	0.9964	0.9979	**<0.001**
INR	1.074	0.9798	1.1789	0.126
CRP	0.999	0.9979	1.0003	0.151
*Model 2.2*				
PLR	0.999	0.9996	1.000	0.678
Age	1.022	1.0156	1.029	**<0.001**
Gender	0.883	0.762	1.025	0.102
BMI	0.999	0.9884	1.01	0.904
WBC	1.008	1.0052	1.012	**<0.001**
Charlson comorbidity index	1.050	1.019	1.083	0.001
Platelet	0.997	0.9964	0.998	**<0.001**
INR	1.118	1.0139	1.233	0.023
CRP	0.999	0.9981	1.000	0.223

BMI, body mass index; WBC, white blood cell; INR, international normalized ratio; CRP, C-reactive protein; PLR, platelet to lymphocyte ratio; NLR, neutrophil to lymphocyte ratio.

### 3.3 RCS curves and Kaplan–Meier curves

**Figure 3:**
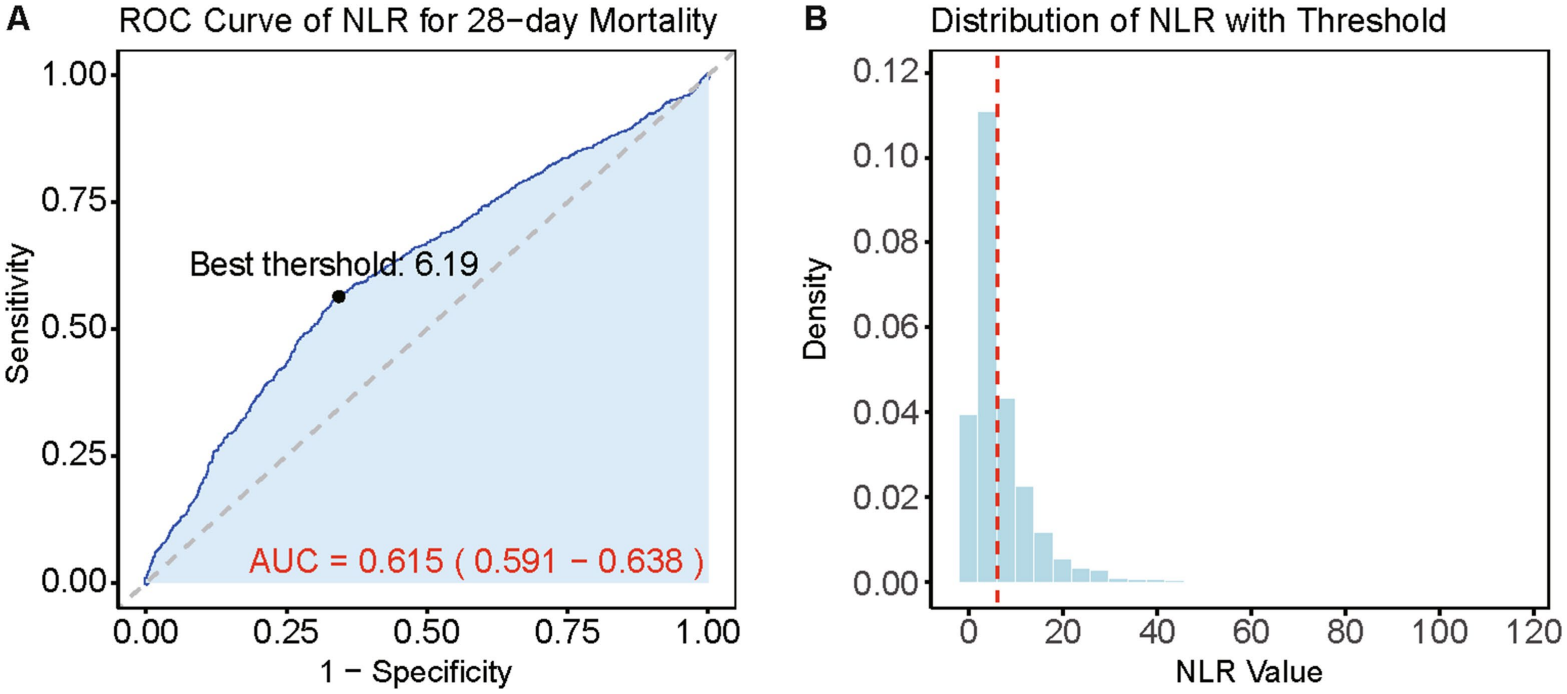
Best threshold selection for NLR and its relationship in NLR distribution analysis. **(A)** ROC curve for the NLR in predicting 28-day mortality. The best threshold and the curve of AUC and 95% CI is highlighted. **(B)** Distribution of NLR values with the best threshold (red dashed line).

**Figure 4:**
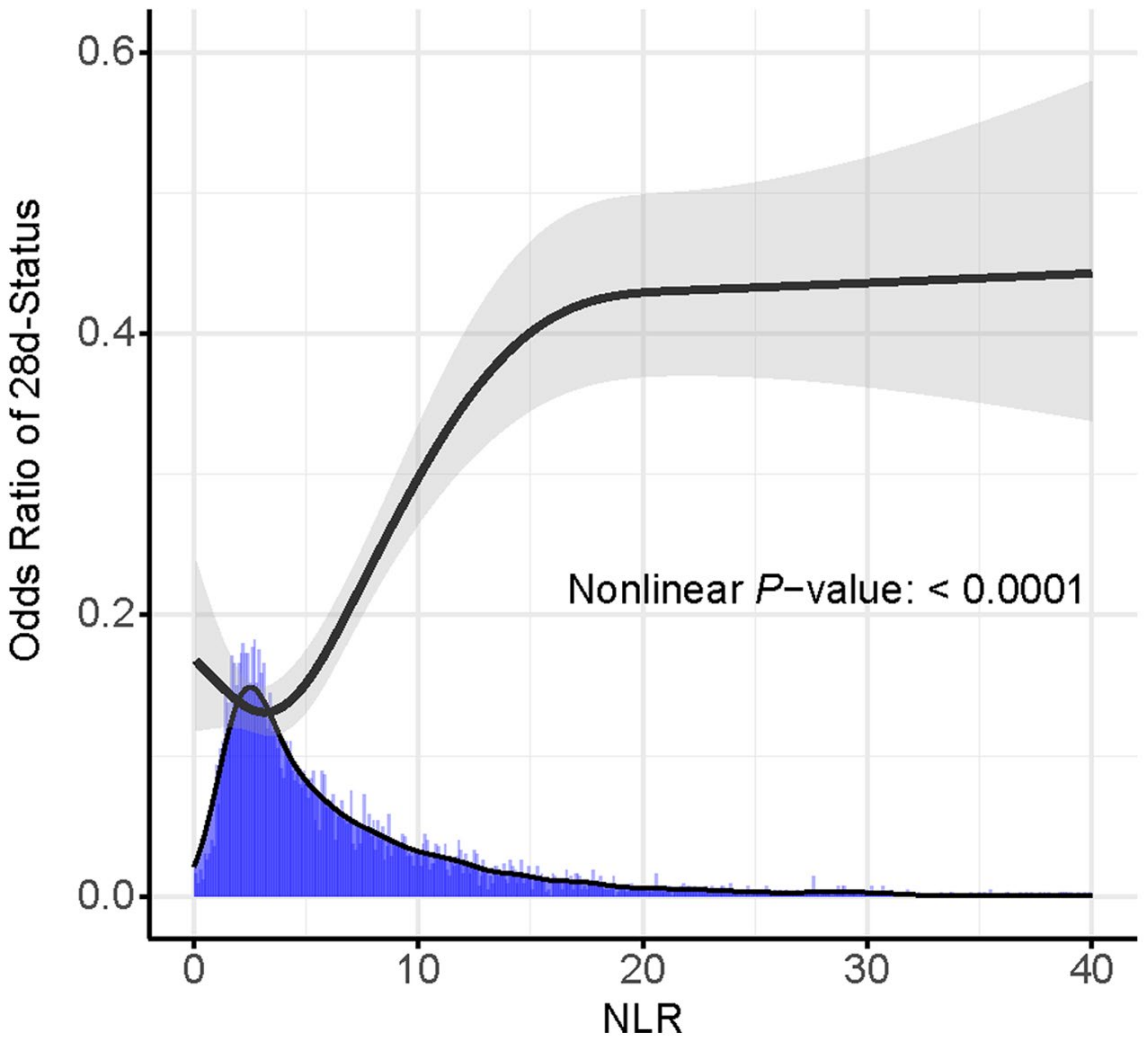
RCS curve for NLR with 95% CI shown as shaded ribbons.

**Figure 5:**
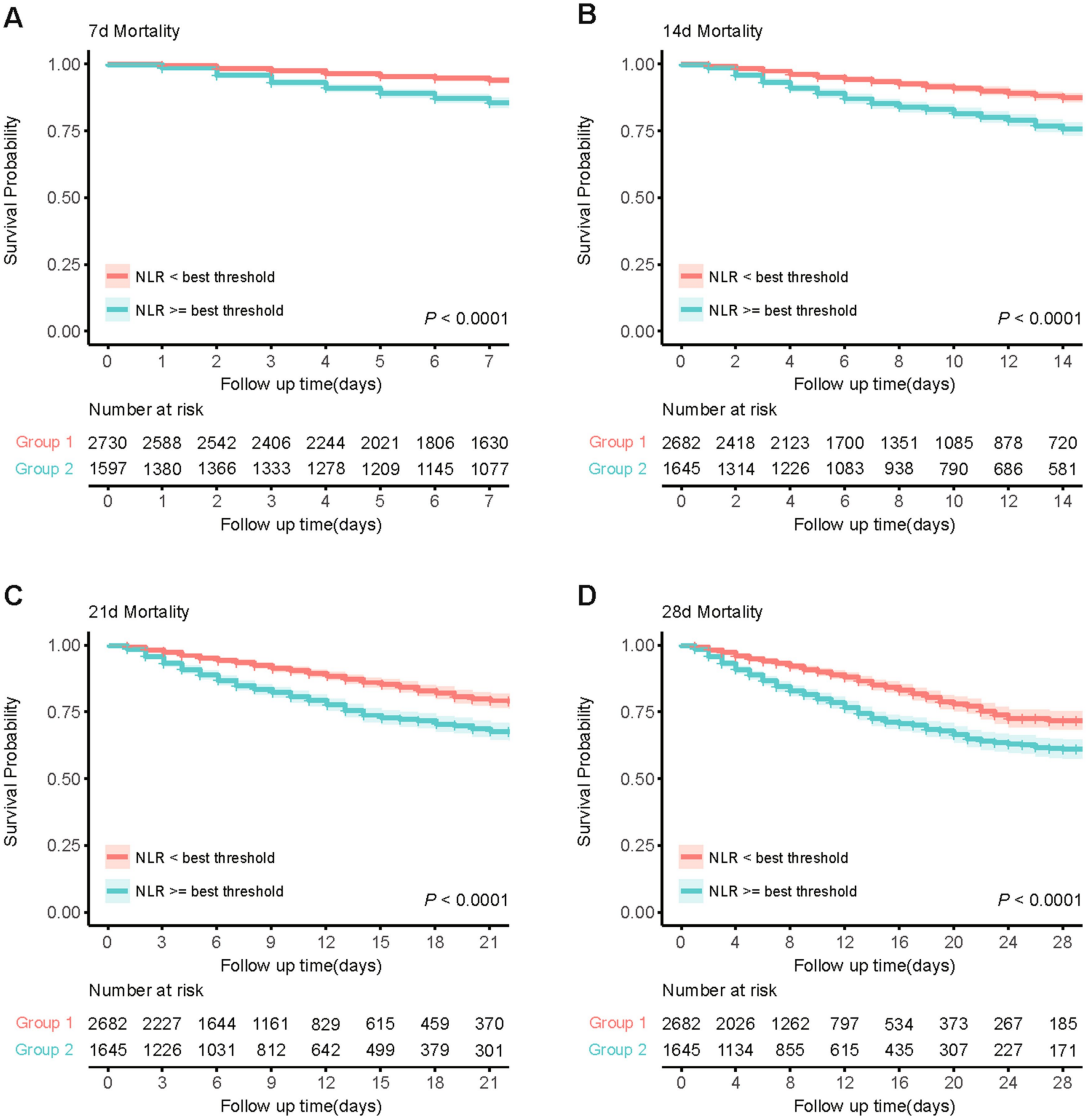
Kaplan–Meier survival analysis curves for short-term all-cause mortality. **(A)** Kaplan-Meier curve for 7-day mortality based on NLR threshold. **(B)** Kaplan-Meier curve for 14-day mortality based on NLR threshold. **(C)** Kaplan-Meier curve for 21-day mortality based on NLR threshold. **(D)** Kaplan-Meier curve for 28-day mortality based on NLR threshold.

Based on the Youden’s index, we determined the best threshold value to stratify CVD patients into a high NLR group (NLR ≥ 6.19, *n* = 1,645) and a low NLR group (NLR < 6.19, *n* = 2,682). Figure 3 illustrates the selection of the best threshold maximizing the risk ratio and the association between NLR ≥ 6.19 and the distribution of NLR. Restricted cubic spline (RCS) analysis was employed to investigate the nonlinear association between NLR and 28-day mortality in CVD subjects (Figure 4). Kaplan–Meier survival curves (Figure 5) demonstrate that the high NLR group exhibited a significantly higher mortality rate compared to the low NLR group over short-term periods of 7-, 14-, 21-, and 28-days (all *p* < 0.001).

### 3.4 Subgroup analysis and forest plots

**Figure 6:**
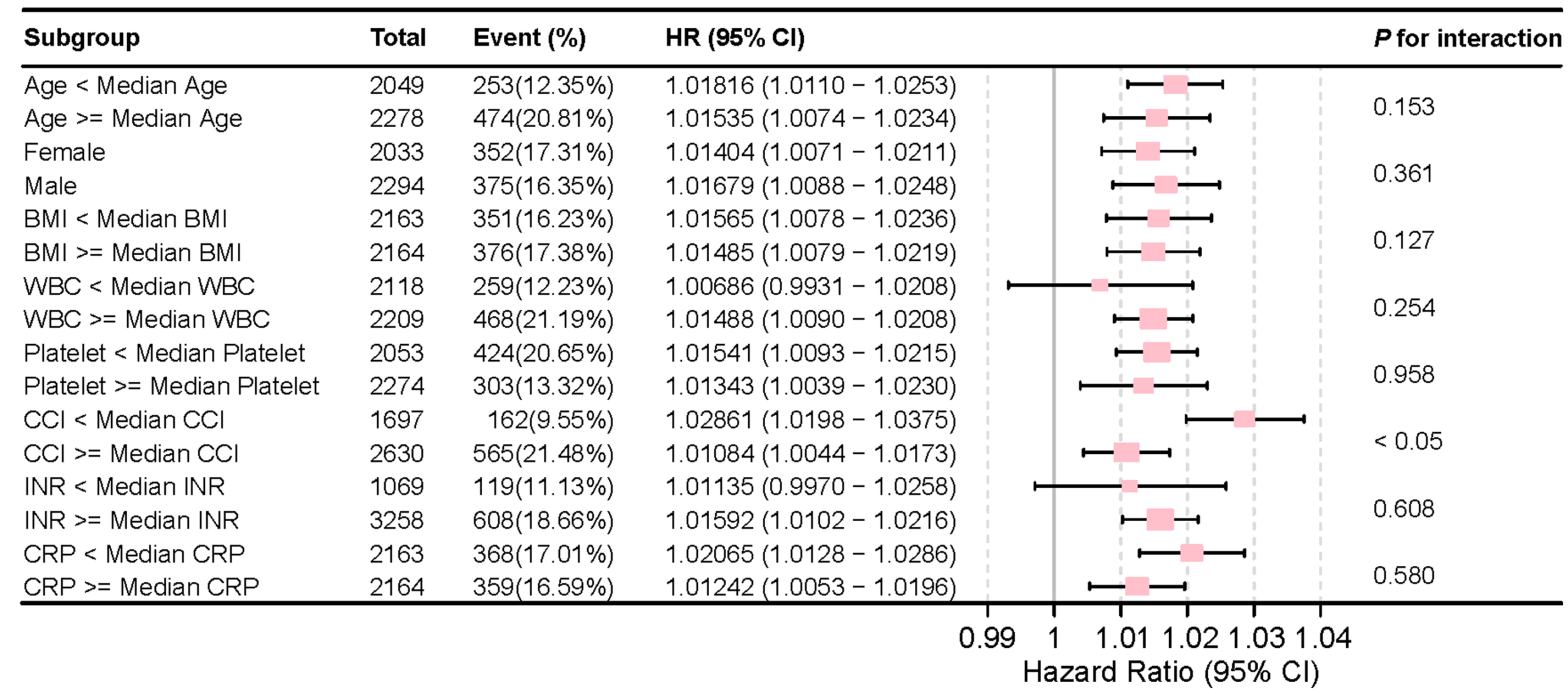
Forest plot for the subgroup analysis of the relationship between hospital mortality and NLR.

Figure 6 illustrates the stable correlation between NLR and the risk of all-cause mortality within 28 days of admission in patients with CVD, ensuring consistency across different subgroups. The meticulously constructed forest plot from subgroup analysis considers variables such as age, gender, BMI, WBC counts, CCI, platelet counts, INR, and CRP. The analysis reveals that NLR, except for an interaction with CCI (*p* < 0.05), did not exhibit significant interactions with any other subgroup variables, with *p*-value for interaction ranging from 0.127 to 0.958. The absence of significant interactions underscores the independence of NLR as a prognostic factor, indicating its association with mortality risk is not confounded by considered demographic and other laboratory covariates.

## 4 Discussion

Our study included 4,327 patients from the MIMIC-IV (v2.2) database. The results of this retrospective study indicate that NLR is an independent factor for all-cause mortality within 28 days of admission for patients with CVD in the ICU. Statistical analysis revealed significantly elevated NLR in patients with CVD who died within 28 days of ICU admission. Further Cox regression analysis was conducted to identify independent predictors of 28-day mortality, adjusting for confounding factors before and after analysis. NLR was found to be independently associated with 28-day mortality compared to other variables. Additionally, we identified the optimal threshold point (best threshold = 6.19), enabling us to construct Kaplan–Meier curves that demonstrated NLR effectively distinguishing patients who died within 28 days, as well as within other short-terms including 7-, 14-, and 21- days. Furthermore, after adjusting for all confounding factors and creating forest plots, NLR emerged as a stable indicator unaffected by other variables, particularly platelet counts, and INR values more closely related to CVD. Therefore, NLR is a reliable predictor of short-term mortality in ICU patients with CVD and can be used as a novel clinical biomarker.

Inflammation is a central component of the immune response and plays a crucial role in cerebrovascular diseases. It can lead to vascular endothelial cell damage, platelet activation, and thrombosis, promoting the development of atherosclerosis and increasing the risk of cerebrovascular events. Neutrophils, T cells, and B cells are key players in this process, contributing to inflammatory responses and immune regulation through cytokine and chemokine secretion. Atherosclerosis, the primary pathological basis of CVD, is driven by the accumulation of lipids and inflammatory cells within vascular walls, which elevates the risk of strokes and other cerebrovascular complications (13). Neutrophils, as key components of innate immunity, exacerbate endothelial dysfunction and plaque instability through the release of cytokines and extracellular traps, thereby promoting the progression of atherosclerosis. In contrast, lymphocytes, especially T cells, exert anti-inflammatory effects by modulating cytokine levels and stabilizing plaques (14).

In stroke patients, neutrophil counts are significantly elevated, with higher counts observed in deceased patients (15). During the acute phase, neutrophil counts increase while lymphocyte counts decrease; in the subacute phase, monocyte counts increase; and in the chronic phase, lymphocyte counts increase while neutrophil counts decrease (16). In ischemic stroke, low lymphocyte counts appear to increase the risk of hemorrhagic transformation (17). Indeed, one study showed that low lymphocyte levels were independently correlated with stroke severity (18). In our study, by employing the NLR, we mitigate the influence of single factors on regulatory mechanisms through reverse changes in two distinct mechanisms, thereby more accurately predicting outcomes in critically ill cerebrovascular disease patients.

NLR is a novel composite biomarker reflecting inflammatory status, calculated by dividing absolute neutrophil counts by lymphocyte counts from routine peripheral blood tests. This ratio is both accessible and cost-effective, integrating critical information from two key white blood cell subtypes. It also minimizes potential biases from absolute values that may fluctuate due to infections, dehydration, or other conditions (19). Increased NLR correlates closely with the occurrence of inflammatory responses, where elevated neutrophil counts reflect the intensity of inflammation and decreased lymphocyte counts indicate physiological stress. Clinically, NLR has been shown to be closely associated with the prognosis of various diseases, including but not limited to chronic obstructive pulmonary disease, ulcerative colitis, gastrointestinal stromal tumors, atherosclerosis, liver cancer, and pancreatic cancer (19–22). NLR also aids in early identification of severe stroke-associated pneumonia (SAP) and predicting ICU admission (23). Studies show that NLR and PLR can predict clinical outcomes in acute ischemic stroke (AIS) patients (24, 25) and hemorrhagic transformation (17, 26). Notably, high NLR levels 24 h post-mechanical thrombectomy are associated with worse functional outcomes, and high NLR predicts hemorrhagic transformation after stroke (24, 27–29). In large vessel occlusion stroke, high NLR correlates with early neurological deterioration and post-stroke depression (30, 31). Clinically, high platelet CD84 expression levels were associated with poor outcomes in stroke patients (32). However, PLR is generally inferior to NLR in predicting outcomes and complications such as edema, and its role in intracerebral hemorrhage (ICH) is limited (33, 34). Only a retrospective study showing that high platelet levels (>100) in ICU-admitted patients correlated with worse GCS scores (35). Tufan’s study found that only high NLR correlates with mortality in stroke patients, whereas PLR and lymphocyte-to-monocyte ratio (LMR) do not (36). Reznik’s study indicated that early NLR elevation predicts delayed-onset delirium (37). Xu et al. found a significant association between diabetes mellitus and NLR with stroke progression and functional outcomes (38). Elevated NLR in spontaneous intracerebral hemorrhage is linked to poor prognosis and disability at 30 days and 3 months (39). In summary, the analysis showed that: (1) NLR levels in patients with vulnerable carotid plaques were significantly elevated compared with other patients; (2) NLR levels were positively correlated with the vulnerability of carotid plaques; and (3) Elevated NLR increased the risk of vulnerable carotid plaques in patients with acute ischemic stroke. NLR provides valuable information on the integrated inflammatory activity and plaque burden in atherosclerosis (40).

More importantly, NLR is crucial in atherosclerosis, influencing plaque formation, development, and prognosis (7, 41). An early rise in NLR values can predict perihemorrhagic edema, infection risk, and early neurological deterioration (39). Hence, monitoring inflammatory indicators regularly is vital for clinicians to assess disease progression and make timely decisions.

In atherosclerosis, neutrophils contribute to plaque initiation and progression through endothelial dysfunction, foam cell formation, plaque instability, and other mechanisms. Increased neutrophils in stroke patients are associated with worse outcomes (42). Neutrophils migrate to the brain early, causing granular tissue formation and secondary damage through inflammatory mediators (43–45). Neutrophils exacerbate ischemic injury by adhering to the endothelium, producing proteases and ROS, and releasing eicosanoids and other substances that damage blood vessels and brain tissue (46, 47). Additionally, neutrophils release extracellular traps, inducing macrophages to release extracellular cytokines and damage-associated molecular patterns, activating Th-17 cells, further which amplifying immune cell recruitment within atherosclerotic plaques, thereby contributing to plaque instability and rupture (48, 49).

In contrast, lymphocytes’ anti-inflammatory characteristics help stabilize plaques and mitigate inflammation. Lymphocytes have been reported to upregulate anti-inflammatory cytokines such as interleukin (IL)-10 and suppress inflammatory cytokines including tumor necrosis factor (TNF)-*α* and IL-6, exerting anti-inflammatory effects (50). In other studies, the protective role of B lymphocytes in the formation of atherosclerosis has been confirmed (51, 52). In parallel, T cells have been described as key drivers and regulators of the pathogenesis of atherosclerosis, with Th1 cells promoting atherogenic features and Treg cells exerting anti-atherogenic effects (53). Interaction of T Lymphocytes with platelets may also have hemostatic effects preventing hemorrhagic transformation after severe ischemic stroke (46). Treg cells, known as immunosuppressive factors, are decreased in IS and are involved in poor stroke outcomes. In experimental stroke, activation of Treg cells in the ischemic region might exert neuroprotective effects. Additionally, Treg cells inhibit the production of proinflammatory cytokines and promote the M1 shift to the M2 phenotype associated with IL-10 signaling. Recent studies suggest that IL-2 delivery increases brain-resident Treg cells and protects against neuroinflammation (47).

Similarly, as inflammatory markers, CRP is synthesized in the liver in response to IL-6 secretion by macrophages and T-cells, is more sensitive and accurately detects low-grade inflammation (54). However, CRP levels can increase in IS and various other inflammatory conditions, reflecting its poor specificity and sensitivity (55). This aligns with our findings. Platelet counts and size reflect platelet activity and serves as a predictive and prognostic biomarker of cardiovascular events. Increased MPV has been observed in cardiovascular diseases (33). Our results showed that platelet count was associated with mortality and survival time in ICU patients with CVD. In our study, PLR was related to these patients in univariate Cox regression but was inferior to NLR as a predictor of outcomes and severe neurological complications such as edema in a larger cohort study (34). Consistently, the association of PLR was not significant in multivariate Cox regression in our findings. NLR and PLR, even with AUC values below 0.7, can still be significant for specific patient subgroups, especially when combined with other clinical indicators. Studies show that NLR and PLR can assist clinicians in evaluating patient condition changes and inflammatory status, facilitating timely treatment adjustments, such as in cases of septic shock and acute coronary syndrome (56, 57). They may also provide higher clinical value for specific populations, such as the elderly or those with multiple chronic conditions, despite an overall lower AUC. Thus, NLR serves not only as an inflammatory marker but also potentially as a potent tool for assessing atherosclerotic progression and predicting cardiovascular events.

NLR can consistently predict the risk of 28-day all-cause mortality in various subgroups, including subgroups based on age, gender, BMI and other clinical parameters. The lack of significant interactions between NLR and most of these variables (except CCI) confirms the strong prognostic value of NLR, which is not affected by other demographic and laboratory covariates.

The significant interaction with CCI suggests that although NLR remains a strong prognostic indicator, its predictive ability may be affected by the level of comorbidities, highlighting the need for a comprehensive risk assessment strategy in clinical practice. These findings emphasize the importance of NLR as an important indicator for predicting mortality risk, even after adjusting for other clinical factors. Our study demonstrates NLR as an independent predictor of short-term all-cause mortality in ICU-admitted CVD patients. Compared to single WBC counts and traditional inflammatory markers like CRP, NLR exhibits more accurate prognostic capabilities. Independent of traditional risk factors, this further underscores its potential to provide information for personalized therapeutic approaches in managing adverse patient outcomes.

However, our study has limitations. Firstly, its cross-sectional nature restricts establishing causality between NLR and cerebrovascular outcomes. Future prospective studies should incorporate comprehensive inflammatory markers and encompass diverse treatment regimens among cerebrovascular disease patient cohorts to further validate NLR’s predictive role and elucidate its mechanisms in disease progression. Secondly, unrecorded medications and hospital nursing practices that may affect cerebrovascular disease could bias our results. Nonetheless, our study positions NLR as a robust biomarker for assessing short-term prognosis in ICU patients with cerebrovascular disease, promising in enhancing predictive accuracy and guiding tailored intervention strategies. Thorough validation through future prospective research is crucial for confirming NLR’s reliability and expanding its application across the entire field of cerebrovascular diseases.

## 5 Conclusion

In conclusion, our study suggests that NLR may serve as an independent predictor of short-term all-cause mortality in ICU patients with CVD. These findings indicate that NLR could be a useful tool for healthcare providers to aid in the assessment of disease severity and prognosis. Incorporating NLR into clinical practice might offer additional support for diagnosing and managing CVD patients, potentially improving the identification of those at higher risk and facilitating timely interventions.

## Data Availability

The datasets presented in this study can be found in online repositories. The names of the repository/repositories and accession number(s) can be found below: MIMIC-IV database (v2.2) https://physionet.org/content/mimiciv/2.2/.

## Footnotes

^1^https://physionet.org/content/mimiciv/2.2/

^2^https://physionet.org/

## Glossary

NLR: Neutrophil-to-Lymphocyte Ratio
CVD: Cerebrovascular Disease
ICU: Intensive Care Unit
MIMIC-IV: Medical Information Mart for Intensive Care (version 4)
K-M: Kaplan–Meier
ROC: Receiver Operating Characteristic
AUC: Area Under the Curve
RCS: Restricted Cubic Spline
BMI: Body Mass Index
CCI: Charlson Comorbidity Index
WBC: White Blood Cell
INR: International Normalized Ratio
CRP: C-Reactive Protein
HR: Hazard Ratio
CI: Confidence Interval
PLR: Platelet-to-Lymphocyte Ratio
IQR: Interquartile Range
BUN: Blood Urea Nitrogen
PT: Prothrombin Time
MPV: Mean Platelet Volume
IL: Interleukin
TNF: Tumor Necrosis Factor
SAP: Stroke-associated Pneumonia
AIS: Acute Ischemic Stroke
VIF: Variance Inflation Factor
