# Supplementary material for: Association between neutrophil-to-lymphocyte ratio and short-term all-cause mortality in patients with cerebrovascular disease admitted to the intensive care unit-a study based on the MIMIC-IV database
Source: Front Med (Lausanne). 2024 Oct 2;11:1457364. doi: 10.3389/fmed.2024.1457364 (PMC11480710; doi:10.3389/fmed.2024.1457364)
Supplement: Supplementary file 1 [file Table_1.DOCX]

**Supplementary Table 1. Tests of Normality for all Variance**

| **Tests of Normality** | | | | | | |
| --- | --- | --- | --- | --- | --- | --- |
|  | **Kolmogorov-Smirnov^a^** | | | **Shapiro-Wilk** | | |
|  | **Statistic** | **df** | **Sig.** | **Statistic** | **df** | **Sig.** |
| **Age** | .053 | 4322 | <.001 | .971 | 4322 | <.001 |
| **Height** | .053 | 4322 | <.001 | .976 | 4322 | <.001 |
| **Weight** | .065 | 4322 | <.001 | .942 | 4322 | <.001 |
| **BMI** | .076 | 4322 | <.001 | .927 | 4322 | <.001 |
| **SBP** | .041 | 4322 | <.001 | .987 | 4322 | <.001 |
| **DBP** | .062 | 4322 | <.001 | .982 | 4322 | <.001 |
| **Heart Rate** | .068 | 4322 | <.001 | .969 | 4322 | <.001 |
| **GCS** | .388 | 4322 | <.001 | .467 | 4322 | <.001 |
| **WBC** | .166 | 4322 | <.001 | .502 | 4322 | <.001 |
| **Lymphocytes** | .359 | 4322 | <.001 | .115 | 4322 | <.001 |
| **Monocytes** | .147 | 4322 | <.001 | .693 | 4322 | <.001 |
| **Neutrophil** | .107 | 4322 | <.001 | .841 | 4322 | <.001 |
| **RBC** | .031 | 4322 | <.001 | .992 | 4322 | <.001 |
| **Platelet** | .098 | 4322 | <.001 | .914 | 4322 | <.001 |
| **D-Dimer** | .251 | 4322 | <.001 | .625 | 4322 | <.001 |
| **Fibrinogen** | .105 | 4322 | <.001 | .906 | 4322 | <.001 |
| **INR** | .297 | 4322 | <.001 | .486 | 4322 | <.001 |
| **PT** | .275 | 4322 | <.001 | .444 | 4322 | <.001 |
| **APTT** | .266 | 4322 | <.001 | .570 | 4322 | <.001 |
| **CRP** | .253 | 4322 | <.001 | .686 | 4322 | <.001 |
| **PLR** | .204 | 4322 | <.001 | .579 | 4322 | <.001 |
| **NLR** | .212 | 4322 | <.001 | .604 | 4322 | <.001 |
| a. Lilliefors Significance Correction | | | | | | |
