# Supplementary material for: Association between neutrophil-to-lymphocyte ratio and short-term all-cause mortality in patients with cerebrovascular disease admitted to the intensive care unit-a study based on the MIMIC-IV database
Source: Front Med (Lausanne). 2024 Oct 2;11:1457364. doi: 10.3389/fmed.2024.1457364 (PMC11480710; doi:10.3389/fmed.2024.1457364)
Supplement: Supplementary file 2 [file Table_2.DOCX]

**Supplementary Table 2. Variance Inflation Factor (VIF) for Covariates in the Cox Regression Models**

| **Model** | **Variable** | **VIF** |
| --- | --- | --- |
| ***Model 1.1 (NLR)*** | NLR | 1.00377129 |
|  | Age | 1.01362033 |
|  | Gender | 1.0147253 |
|  | BMI | 1.00256075 |
| ***Model 1.2*** | PLR | 1.00197787 |
|  | Age | 1.01379494 |
|  | Gender | 1.01267019 |
|  | BMI | 1.0003902 |
| ***Model 2.1*** | NLR | 1.05047342 |
|  | Age | 1.37297646 |
|  | Gender | 1.04466292 |
|  | BMI | 1.00487085 |
|  | WBC | 1.02244904 |
|  | Charlson comorbidity index | 1.38269973 |
|  | Platelet | 1.0440854 |
|  | INR | 1.06817631 |
|  | CRP | 1.00826257 |
| ***Model 2.2*** | PLR | 1.12232559 |
|  | Age | 1.3631611 |
|  | Gender | 1.03840762 |
|  | BMI | 1.00276299 |
|  | WBC | 1.03158323 |
|  | Charlson comorbidity index | 1.36700734 |
|  | Platelet | 1.13950524 |
|  | INR | 1.03378717 |
|  | CRP | 1.00652824 |
